# Diagnosis and management of intercostal intramuscular hemangioma: an updated review

**DOI:** 10.1186/s13019-023-02328-9

**Published:** 2023-07-04

**Authors:** Takahiro Ochi, Yasuo Sekine, Eitetsu Koh, Hidehisa Hoshino, Tadao Nakazawa

**Affiliations:** 1 Department of Thoracic Surgery, Tokyo Women’s Medical University Yachiyo Medical Center, 477-96, Owada-Shinden, Yachiyo, 276-8524 Chiba Japan; 2 Department of Pathology, Tokyo Women’s Medical University Yachiyo Medical Center, 477-96, Owada-Shinden, Yachiyo, 276-8524 Chiba Japan

**Keywords:** Intercostal intramuscular hemangioma, Intramuscular hemangioma, Chest wall tumor

## Abstract

**Background:**

Intramuscular hemangioma (IMH) is an uncommon type of hemangioma, and primary IMH of the intercostal muscle is even rarer. Only a few reports describe IMH of the intercostal muscle, and there are no review articles on this topic. We report our experience with a younger female patient, who underwent video-assisted thoracic surgery with tumor resection and review the previous literatures of intercostal IMH.

**Case presentation:**

An asymptomatic 17-year-old woman showed a 29-mm, homogeneous, intrathoracic nodule in the left chest wall, attached to the second and third ribs on computed tomography. We performed exploratory thoracoscopic surgery and the tumor was excised without surrounding rib resection. Histopathologic examination of the surgical specimen revealed proliferation of small blood vessels within the surrounding striated muscle, leading to the diagnosis of intercostal IMH. The surgical margin was negative. The patient’s postoperative course was uneventful, and there has been no evidence of recurrence for more than 18 months after surgery.

**Conclusions:**

We describe a case of intercostal IMH, who received tumor resection with clear excision margin without surrounding rib resection. Preoperative diagnosis is challenging due to its rarity, but intercostal IMH should be recalled as a differential diagnosis of chest wall tumor. Tumor excision without surrounding rib resection is acceptable for intercostal IMH, when there is a good possibility of achieving negative surgical margin.

## Background

Intramuscular hemangioma (IMH) is an uncommon type of hemangioma, and primary IMH of the intercostal muscle is even rarer. Only a few reports describe IMH of the intercostal muscle, and there are no review articles on this topic.

We describe herein our experience with a younger female patient with IMH arising from the intercostal muscle, who underwent video-assisted thoracic surgery with tumor resection; we were able to achieve negative margin without resecting the surrounding ribs. We also provide a review of the English-language literature on the subject of intercostal IMH, to elucidate the clinicopathological characteristics.

**Fig. 1:**
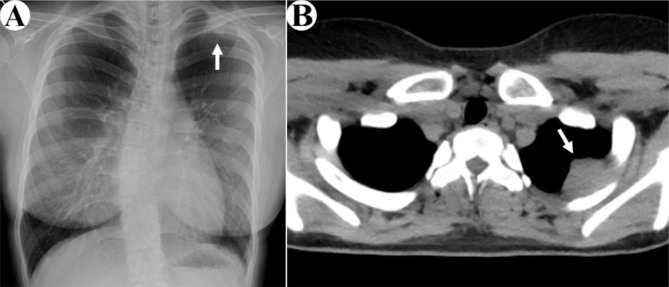
Preoperative radiological imaging. **a**: Chest radiography shows an abnormal shadow in the left upper lung field, representing the extrapleural sign (white arrow). **b**: Chest computed tomography shows a 29-mm nodule in the left second intercostal space (white arrow)

## Case presentation

An asymptomatic 17-year-old woman was referred to our institution when an abnormality on chest radiography was noted during her routine medical check-up. The x-ray showed a nodular shadow at the left upper lung field (Fig. 1a). Chest computed tomography revealed a 29-mm, homogeneous, intrathoracic nodule in the left chest wall, attached to the second and third ribs (Fig. 1b). At this stage, we suspected a neurogenic tumor but we decided to perform exploratory surgery because of the possibility of malignancy without additional close examination, according to the patient’s and her family’s intention.

**Table 1:** Prior English-language reports of intercostal intramuscular hemangiomas: patient characteristics

Source	Age, y	Sex	Localization	Size, cm	Relation to rib cage	Correct preoperative diagnosis	Resection including the surrounding ribs	Pathological classification	Follow-up, mo	Recurrence
Winchester, 1992^5)^	39	F	Right fifth	5.0	Inside	No	Yes	Small vessel	unknown	unknown
Ono, 1996^6)^	33	M	Right fourth	9.5	Inside	No	No	Mixed	36	None
Kara, 2000^7)^	46	M	Right second	4.0	Inside	No	No	Large vessel	48	None
Yonehara, 2000^8)^	33	M	Left sixth	5.0	Outside	No	Yes	Small vessel	60	None
Kubo, 2004^9)^	27	M	Right seventh	5.5	Outside	Yes	Yes	Large vessel	6	None
Ulku, 2010^10)^	11	F	Right nineth-eleventh	8.5	Outside	Yes	Yes	Large vessel	6	None
Elbawab, 2019^11)^	14	M	Right fifth	6.5	Inside	No	Yes	Large vessel	6	None
Dantis, 2021^12)^	18	M	Left seventh	4.2	Outside	No	No	Large vessel	12	None
This case, 2022	17	F	Left second	2.9	Inside	No	No	Small vessel	18	None

F: female; M: male

**Fig. 2:**
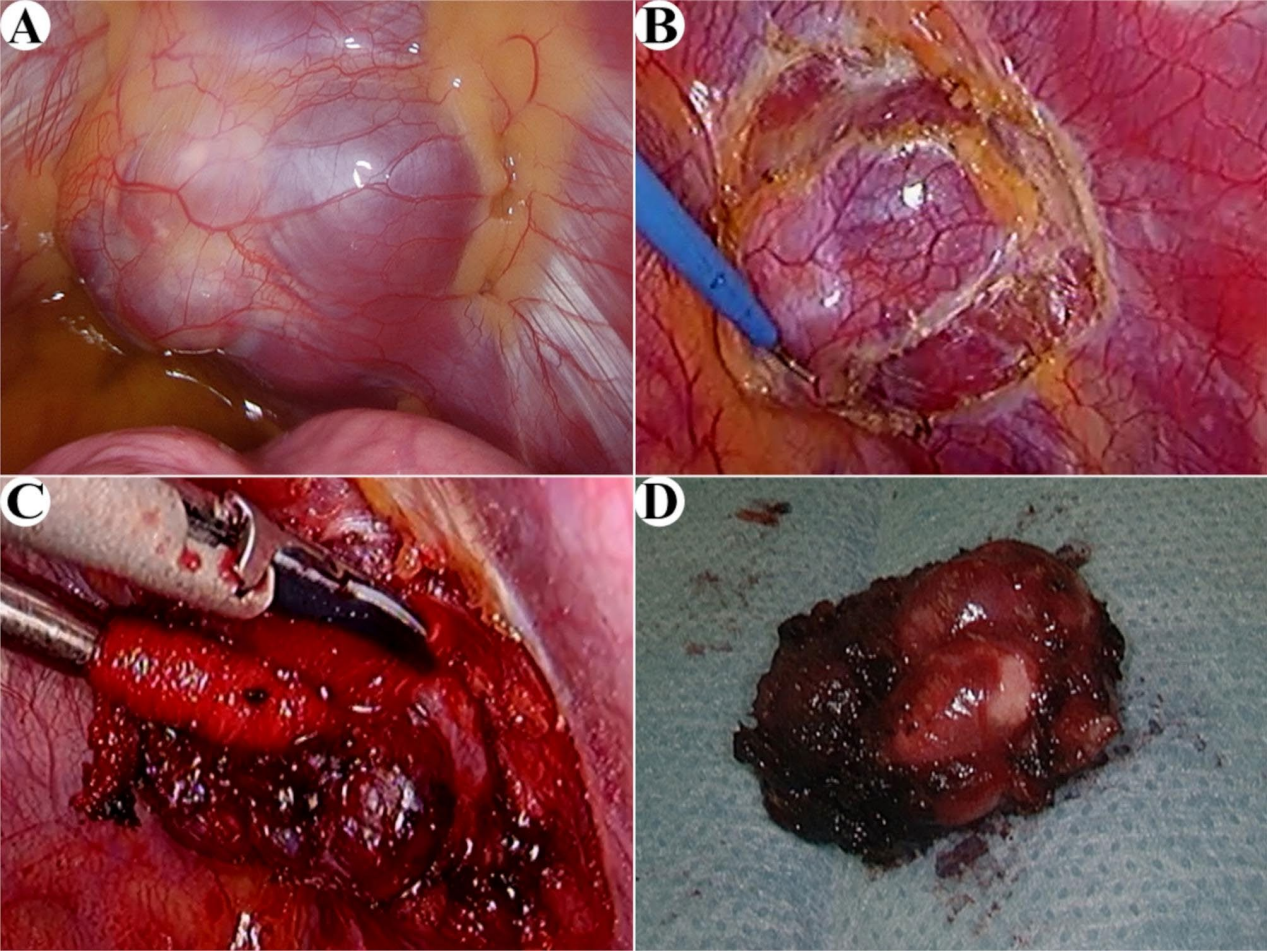
Intraoperative findings **a**: The uneven solid tumor is arising from the left second intercostal space. **b**: The surrounding parietal pleura was incised. **c**: The tumor was detached from the intercostal muscles. **d**: Macroscopic findings of surgical specimen

**Fig. 3:**
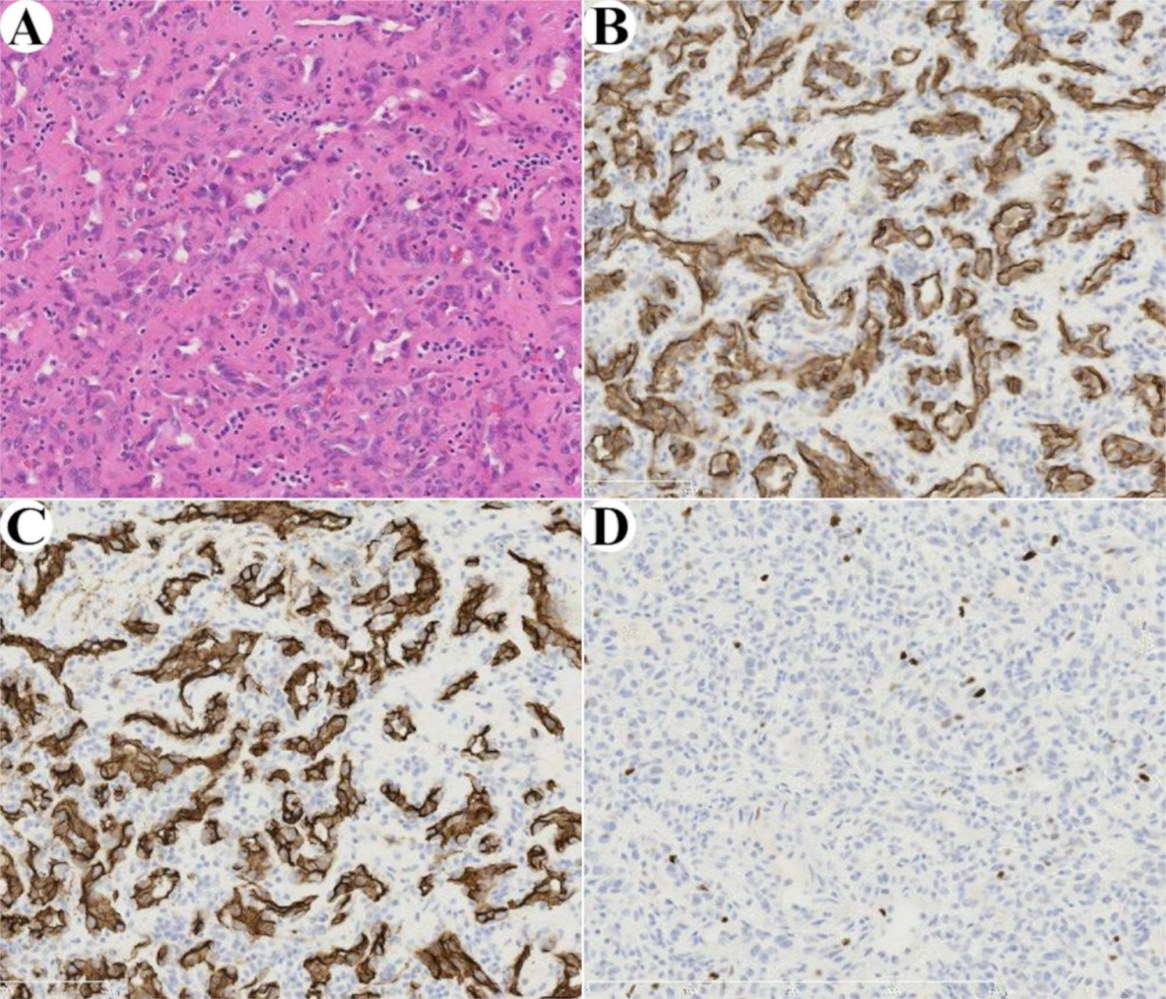
Histopathological findings of surgical specimen. Hematoxylin-eosin staining reveals proliferation of small blood vessels **(a)**. Tumor cells are positive for CD31 **(b)** and CD34 **(c)**. Immunostaining of Ki-67 shows a low proliferative rate (< 1%) **(d)**

**Fig. 4:**
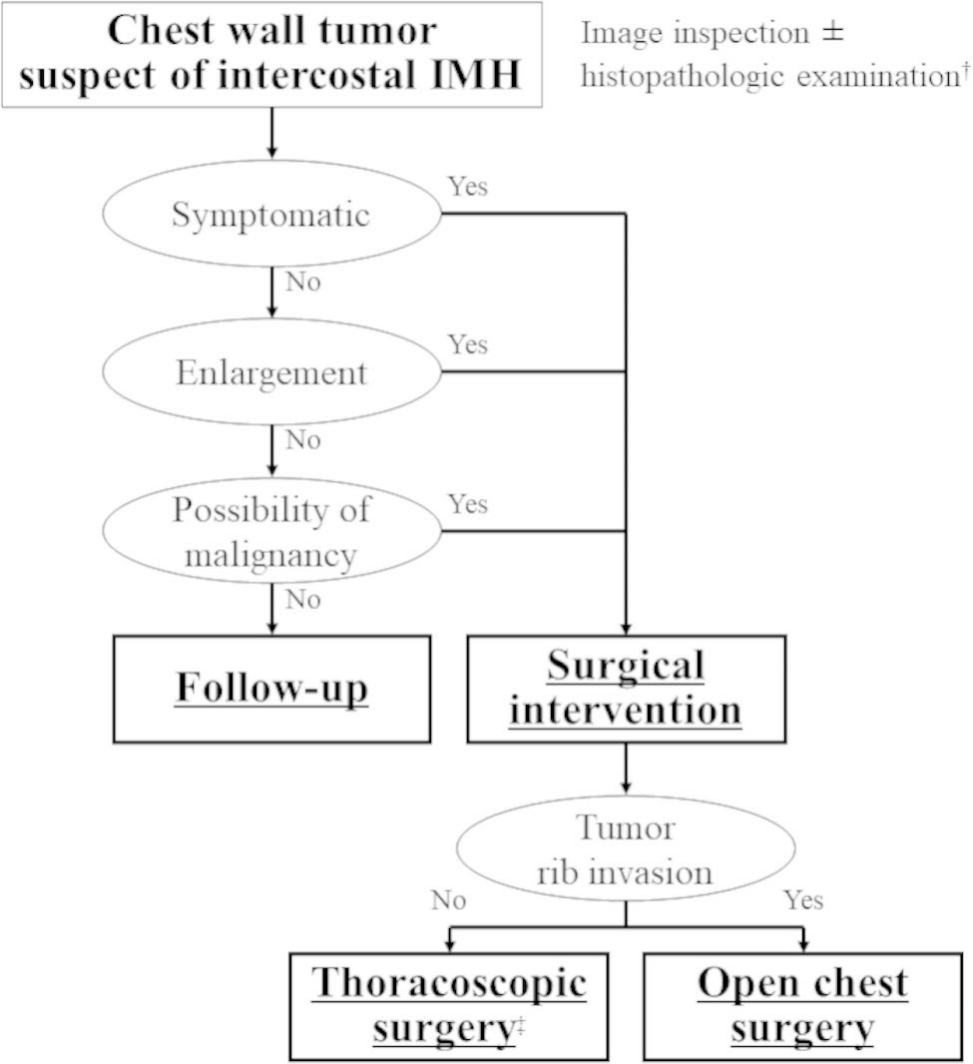
Management flow chart of intercostal intramuscular hemangioma. ^†^If the tumor is suspected to be intercostal intramuscular hemangioma, fine-needle biopsy could be considered, but the risk of bleeding should be noted. ^‡^Video-assisted thoracic surgery and robot-assisted thoracic surgery are included. IMH: intramuscular hemangioma

Three-port video-assisted thoracic surgery was performed using single-lung ventilation, with the patient in the right lateral decubitus position. Skin incisions of 5-mm were made in the second and fourth intercostal space along the anterior axillary line and a 10-mm skin incision was made in the sixth intercostal space along the middle axillary line. The uneven, solid tumor was visualized in the second intercostal space (Fig. 2a). The mass was encapsulated and well-defined under the parietal pleura. There was no visible rib invasion and the tumor could be detached by parietal pleural incision (Fig. 2b). However, the tumor had penetrated the intercostal muscle (Fig. 2c). The intercostal vessels were first excised to reduce bleeding. Thereafter, we dissected the tumor using an ultrasonic scalpel, together with the surrounding intercostal muscle and nerve, to achieve a sufficient margin of excision (Fig. 2d). Because rapid histopathologic diagnosis did not show obvious malignant findings, the surgery was completed without additional resection of the surrounding tissues.

Histopathologic examination of the surgical specimen revealed negative surgical margin and proliferation of small blood vessels within the surrounding striated muscle using hematoxylin-eosin staining (Fig. 3a). Tumor cells were positive for CD31 (Fig. 3b) and CD34 (Fig. 3c). Immunostaining of Ki-67 showed a low proliferative rate (< 1%) (Fig. 3d). The final diagnosis was small-vessel type IMH. The patient’s postoperative course was uneventful, and there has been no evidence of recurrence for more than 18 months after surgery.

## Discussion

The IMH subtype accounts for 0.7% of all hemangiomas [1]. Most patients with IMH are younger than 30 years of age [2], and there is no difference in incidence between male and female patients [3]. The tumor is benign and generally regarded as congenital [1], although it is also suggested that trauma may participate in its pathogenesis [2]. Considering that our patient was young and had no history of trauma, we assume this lesion was congenital. Regarding the management of intercostal IMH, surgical resection is indicated when accompanied by symptoms, when there is a tendency toward enlargement, or when malignancy cannot be ruled out, but follow-up can be considered without these findings (Fig. 4).

In the present study, hematoxylin-eosin staining of the surgical specimen revealed proliferation of small blood vessels, but expression level of Ki-67, proliferation index marker, was low (< 1%). In addition to these, positive immunostaining for CD31 and CD34, blood vessel markers, led to the diagnosis of IMH. Hemangioma is classified into 3 types: small vessel, large vessel, and mixed [4]. Our patient had small-vessel IMH.

IMH is frequently found in the muscles of the upper and lower extremities; intercostal IMH represents 1.2–1.4% of all IMH diagnoses [2, 3]. We identified 8 published articles in which patients were pathologically diagnosed with either intercostal IMH or hemangioma originating in the intercostal muscle (Table 1) [5–12]. In these articles, the mean age at surgery was 27.6 years (range, 11–46 years), and there were only 2 female patients. Most cases had lesions on the right side (75%). The mean tumor size was 6.0 cm (range, 4.0-9.5 cm). Intercostal IMH protruded outward from the ribcage in 4 patients and inward from the ribcage in 4. A correct preoperative diagnosis was made in 2 patients. All patients underwent surgery: 5 underwent extended excision of the tumor with resection of the surrounding ribs, and 3 underwent tumor excision without rib resection. The pathological classification of IMH was as follows: large vessel (n = 5), small vessel (n = 2), and mixed (n = 1). The postoperative course was uneventful in all patients. Of the 7 patients whose postoperative follow-up examinations were discussed, the mean follow-up period was 24.9 months (range, 6–60 months), and no patients had evidence of recurrence during follow-up. Compared with the cases in previous intercostal IMH reports, in the present case, the lesion was discovered at a relatively younger age and the tumor was smaller, which supports the hypothesis that the lesion in this report was congenital.

The preoperative diagnosis of IMH is challenging due to the rarity of this tumor type. One researcher found that a correct preoperative diagnosis was made in only 19% of 335 patients [3]. If the tumor is suspected to be IMH, fine-needle biopsy could be considered, but the risk of bleeding should be noted. The differential diagnosis of chest wall tumor varies widely and includes following diseases: neurogenic tumor, bone tumor, lipoma, desmoid tumor, elastofibroma dorsi, in addition to IMH [13–19]. Extended resection that includes the surrounding tissues could be considered desirable, based on a single report noting that 18% of 76 patients suffered local recurrence after surgical resection [4]. However, other reports note the absence of recurrence after tumor resection with a sufficient margin, even if the surrounding ribs are left intact [6, 7, 12], indicating the possibility of omitting surrounding rib resection if there is no visible involvement of the ribs and the tumor could be detached by parietal pleural incision. For tumor resection of intercostal IMH, thoracoscopic surgery, including video-assisted thoracic surgery and/or robot-assisted thoracic surgery, can be considered. We decided our operative method based on the following factors: size, location and invasiveness of the tumor, and the influence on patient’s appearance and/or function. Our patient was a younger woman with a relatively small, well-defined tumor. We felt confident that we could achieve negative margin and therefore decided against additional excision. We set up a strict follow-up schedule for our patient.

## Conclusion

We describe a case of IMH arising from the intercostal muscle, who received tumor resection with clear excision margin instead of resection of surrounding ribs, and review the previous literatures of IMH of the intercostal muscle. Preoperative diagnosis is challenging due to its rarity, but intercostal IMH should be recalled as a differential diagnosis of chest wall tumor. Tumor excision without surrounding rib resection is acceptable for IMH lesion of the intercostal muscle when there is a good possibility of achieving negative surgical margin.

## Data Availability

The datasets supporting the conclusions of this article are included within the article.

## List of Abbreviations

IMH: Intramuscular hemangioma
